# Association of household fuel with acute respiratory infection (ARI) under-five years children in Bangladesh

**DOI:** 10.3389/fpubh.2022.985445

**Published:** 2022-12-01

**Authors:** Md. Aminul Islam, Mohammad Nayeem Hasan, Tanvir Ahammed, Aniqua Anjum, Ananya Majumder, M. Noor-E-Alam Siddiqui, Sanjoy Kumar Mukharjee, Khandokar Fahmida Sultana, Sabrin Sultana, Md. Jakariya, Prosun Bhattacharya, Samuel Asumadu Sarkodie, Kuldeep Dhama, Jubayer Mumin, Firoz Ahmed

**Affiliations:** ^1^COVID-19 Diagnostic Lab, Department of Microbiology, Noakhali Science and Technology University, Noakhali, Bangladesh; ^2^Advanced Molecular Lab, Department of Microbiology, President Abdul Hamid Medical College, Karimganj, Bangladesh; ^3^Department of Statistics, Shahjalal University of Science and Technology, Sylhet, Bangladesh; ^4^Joint Rohingya Response Program, Food for the Hungry, Cox's Bazar, Bangladesh; ^5^Department of Applied Chemistry and Chemical Engineering, Noakhali Science and Technology University, Noakhali, Bangladesh; ^6^Department of Banking and Insurance, University of Chittagong, Chittagong, Bangladesh; ^7^Department of Environmental Science and Management, North South University, Bashundhara, Dhaka, Bangladesh; ^8^COVID-19 Research, Department of Sustainable Development, Environmental Science and Engineering, KTH Royal Institute of Technology, Stockholm, Sweden; ^9^Nord University Business School (HHN), Bodø, Norway; ^10^Division of Pathology, ICAR-Indian Veterinary Research Institute, Bareilly, Uttar Pradesh, India; ^11^Platform of Medical and Dental Society, Dhaka, Bangladesh

**Keywords:** developing countries, solid fuels, clean fuels, under-five children, acute respiratory infection (ARI)

## Abstract

In developing countries, acute respiratory infections (ARIs) cause a significant number of deaths among children. According to Bangladesh Demographic and Health Survey (BDHS), about 25% of the deaths in children under-five years are caused by ARI in Bangladesh every year. Low-income families frequently rely on wood, coal, and animal excrement for cooking. However, it is unclear whether using alternative fuels offers a health benefit over solid fuels. To clear this doubt, we conducted a study to investigate the effects of fuel usage on ARI in children. In this study, we used the latest BDHS 2017–18 survey data collected by the Government of Bangladesh (GoB) and estimated the effects of fuel use on ARI by constructing multivariable logistic regression models. From the analysis, we found that the crude (the only type of fuel in the model) odds ratio (OR) for ARI is 1.69 [95% confidence interval (CI): 1.06–2.71]. This suggests that children in families using contaminated fuels are 69.3% more likely to experience an ARI episode than children in households using clean fuels. After adjusting for cooking fuel, type of roof material, child's age (months), and sex of the child–the effect of solid fuels is similar to the adjusted odds ratio (AOR) for ARI (OR: 1.69, 95% CI: 1.05–2.72). This implies that an ARI occurrence is 69.2% more likely when compared to the effect of clean fuel. This study found a statistically significant association between solid fuel consumption and the occurrence of ARI in children in households. The correlation between indoor air pollution and clinical parameters of ARI requires further investigation. Our findings will also help other researchers and policymakers to take comprehensive actions by considering fuel type as a risk factor as well as taking proper steps to solve this issue.

## Introduction

Acute respiratory infection (ARI) is the leading cause of death among children under-five years in underdeveloped countries (1). It is a severe infection that makes it difficult to breathe normally (2). Yet, it is nearly impossible to prove whether the main risk factors for developing an ARI from viruses and bacteria originate from the nose, trachea (windpipe), or lungs (3–5). Children, elderly, and people with immune system abnormalities are especially vulnerable (6–9).

Generally, anyone from neonatal to old may be affected by ARI, with diverse clinical symptoms such as runny nose, cough, nasal congestion, sore throat, fatigue, body aches, obstruction, dysphasia, or respiratory distress, accompanied by or without fever (10). The World Health Organization (WHO) confirmed a global alarm for pneumonia called severe acute respiratory syndrome (SARS) on 12 March 2003 (11). ARI causes 15% of the global under-five deaths in children, especially in low- and middle-income countries (LMICs) (12). About 98% of children in LMICs under the age of five are exposed to levels of ambient air pollution that exceed WHO recommendations (13). For children in high-income countries, the figure is 52% (13). Bangladesh is LMIC with almost 166 million people (63%) living in rural regions (14). However, the precise magnitude of ARI which is already doubling at a very large scale in Bangladesh is unknown. Unlike cholera or acute malnutrition, there are no acceptable benchmarks for ARI, making it difficult to measure case management quality using established criteria. Several research studies reported high correlations between environmental risk parameters and developing ARI, such as smoke produced from indoor cooking, different types of outdoor air pollution, passive smoking, and overcrowding (15, 16). These risk factors in children under-five years cause various severe problems, such as low birth weight, malnutrition, measles, pneumonia, and problems in breastfeeding mothers (16). In addition to that, different types of cooking fuel, poor toilet facilities, percentage of mother's literacy, adequate medication for the intestinal parasite, place of residence, body mass index (BMI) of mother and children, and wealth index are also potential risk factors for pneumonia/ARI in developing countries (17–23). Recent studies from India and Guatemala indicated that children born in households using high-polluting solid fuels were 73 and 63 g underweight, respectively, lower in birth weight as compared with children born in households using low-polluting fuels (24, 25).

In rural areas of developing countries, the main risk factors for ARI considered are lack of sanitation system, malnutrition, cooking smoke, and acute respiratory diseases, which are related to the low income and illiteracy (25–27). The burning of cooking fuel is not necessarily the only source of indoor air pollution, although it is considered the major source. Pollutants from dirty contaminated fuel sources used for indoor space heating and lighting are among the other sources (1). Children in the city stay inside their home being exposed to indoor air pollution from biomass fuel due to outdoor space scarcity. Underlying risk factors such as malnutrition among children may also aggravate the problems significantly (28). Because of the scarcity and difficulty in obtaining non-solid or clean fuels such as electricity and natural gas, low-income families in many developing countries rely on the use of low-cost but high-pollution solid fuels, such as wood, coal, straws, and animal dung, as their primary sources of energy for cooking and heating (29–32). Yet, even when access to electricity was available, 19.0, 6.8, and 50.9% of households in Bangladesh used crops, animal dung, and wood, respectively, as fuels for cooking, heating, and lighting (18). The fuels are primarily used in simple, inefficient, and mostly unvented family cooking stoves, resulting in enormous amounts of indoor smoke accumulation due to poor ventilation (33). The number of doors and windows is pertinent in improving house ventilation and is also associated with children's ARI. The odds of ARI in children living in poorly ventilated houses were four times higher than in those living in houses with good ventilation (34).

In Bangladesh, 80% of households use various solid fuel sources for cooking (coal/lignite, charcoal, wood, straw/shrubs/grass, crops, and animal dung), while 20% use clean fuel (electricity and liquid petroleum gas/natural gas/biogas) (35). In addition, the main reason for mortality and morbidity in Bangladesh for diarrhea has been successfully mitigated now, while the relative risk factors for ARI have been increasing day by day (36–39). However, no study to our knowledge has investigated the link between ARI in children and solid fuel exposure in Bangladesh. Thus, using the most up-to-date data available, this study examines the relationship between exposure to solid fuels and ARI in Bangladeshi children under the age of five.

## Materials and methods

### Study area

Bangladesh is a developing country and is one of the most highly densely populated countries in the world, with a delta of rivers that flows down into the Bay of Bengal (40). It is a low-lying, riverine country in South Asia's tropical monsoon region, with an average elevation of 85 meters above sea level and a climate marked by high temperatures, heavy rainfall, cyclones, tidal bores, often excessive humidity, and fairly marked seasonal variations (41, 42).

Bangladesh is a tropical-moist climate-based area characterized by seasonal diversity in precipitation, moderate ambient temperature, and high relative humidity (43). The country has four climatic seasons in a year: lower temperature in the winter (from December to February); higher temperature in the summer (from March to May), the rainy season (from June to September), and the post-monsoon autumn (from October to November) (44). From the observation of daily temperature data in the last 61 years (1960–2021), the average temperature of 25.2°C occurs in July, a minimum temperature of 12.9°C occurs in January while the maximum temperature of 33.5°C is observed in April. We also observed maximum precipitation of 496 mm in July and a minimum of 4 mm in January (43).

### Data source and study design

**Figure 1:**
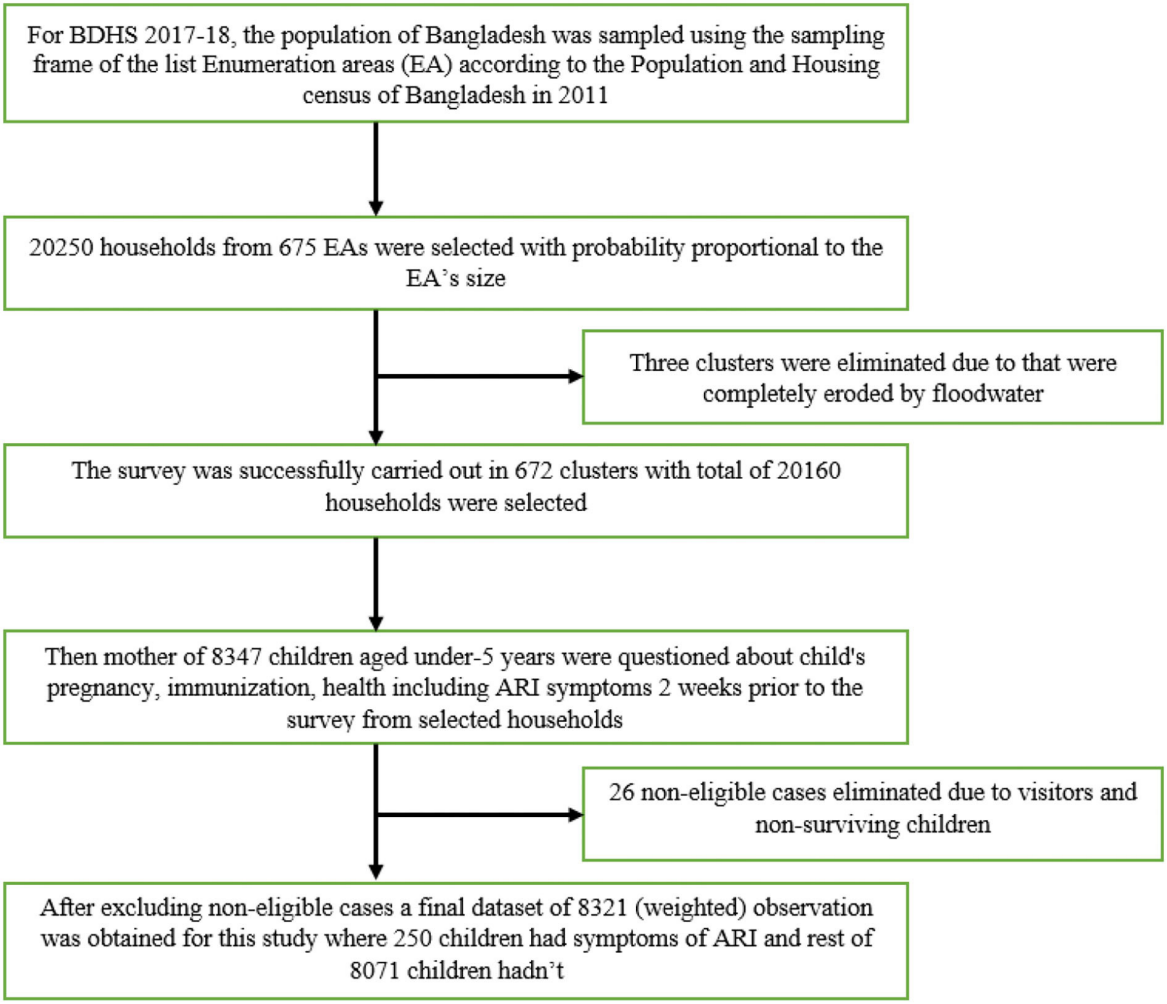
Sample procedure of 2017 BDHS and selection of sample for the study.

This study utilized data from the BDHS 2017–18, a government-organized national survey. In the first step, 675 enumeration areas (EA) were chosen with a probability proportionate to EA size of 250 in urban regions and 425 in rural areas. A calculated sample size of 30 households per EA was selected in the second step of sampling to give statistically credible estimates of key demographic and health characteristics. A total of 20,250 households were selected based on this concept. Since three EAs (two rural and one urban) were severely affected by floodwater, the survey was completed in 672 EAs. We observed that about 20,100 were never-married women aged 15 to 49 who were expected to complete the interviews. Mothers of 8,347 children younger than 5 years were questioned about demographic, economic, pregnancy, postnatal care, immunization, and health issues including ARI symptoms. After limiting our samples to children for whom complete data on the outcome and predictors included in the study were available, we ended up with 8,321 (weighted) children for analysis after eliminating ineligible cases (such as other fuel types, guests, and non-surviving based children) as well as cases with missing survey information on the age of selected children. Figure 1 illustrates the sampling technique. The 2017–18 BDHS report includes a detailed discussion of the sample design and technique. In this study, we used the outcome variable of interest as ARI in children under the age of five. According to 2017–18 BDHS, ARI is known as a mother's or caregiver's acuity whether children with ARI symptoms need to follow proper treatment. In that survey, the ARI symptoms define as, short, rapid breathing that is chest-related and/or difficult breathing. Children under age five with ARI symptoms in the 2 weeks before the survey were included as a sample in this study. The ARI variable was classified as 1, if respondents answered “yes”, or coded as 0 when they responded “no”.

### Exposure variable

The exposure variable is solid fuel, which is determined by the type of fuel used for cooking or heating. Each household's type of cooking fuel is collected by the BDHS. Survey respondents were asked “What type of fuel does your home primarily use for cooking?” (35). Fuel types are classified into coal/lignite, charcoal, wood, straw/shrubs/grass, crops, animal dung, electricity, and liquid petroleum gas/natural gas/biogas. The exposure variable is a binary variable that indicates types of cooking fuel: clean fuel vs. solid fuel. Coal/lignite, charcoal, wood, straw/shrubs/grass, crops, and animal dung are considered solid fuels. The use of electricity and liquid petroleum gas/natural gas/biogas is classified as clean fuels. The fuel type variable is coded as 1 if the household uses clean fuel, otherwise 0 (solid fuel).

### Co-variates

By reviewing the existing literature, the most potentially related and assumed variables associated with ARI were included in this study, such as household-related factors (i.e., place of residence, region, mass media, source of potable water, availability of toilet facility, wealth status, electricity, types of materials for flooring, type of wall material, type of roof material, and the number of household members), parents/caregivers related factors (i.e., having vaccination card, mother's age, education level, BMI, the total count of living children, mother's occupation, mother's work for, household head's occupation, proper education, and type of household head's education), and child-related factors (i.e., child's age, sexual category, time of birth, delivery place, birth weight, C-section delivery, treatment for intestinal diseases, vitamin A supplementation, and nutritional history) (25).

Participants were asked about their history of listening to radio or watching television, and those who replied at least once in 7 days were counted to be routinely exposed to that type of social media (35). Sources of drinking water, supplied piped water sources (piped in dwellings, piped to a yard/plot, public tap water/standpipe water), tube well water (borehole), and other sources of drinking water, were listed (e.g., rainwater and river water, well) (45). Some of the toilet facilities used in this study include improved (flush toilets, flush in a piped sewer system, flush in septic tanks, flush from pit latrines, pit latrines with slabs, and ventilated pit latrines), shared (but improved) toilets with other households, and not advanced (no flush toilet), no flush in the piped sewer facility, no flush in the septic tank (e.g., hanging toilet, open hole) (46). The wealth index was reclassified into upper economic class (above 20% asset value), middle economic class (within 40% asset value), and lower economic class (below 40% asset value) (47). We also observed the main material of the floor/roof/wall of the dwelling. The floor/roof/wall is classified as natural (earth/sand and dung), rudimentary (wood planks and palm/bamboo), and finished (vinyl or asphalt strips, ceramic tiles, cement, and carpet) (35).

The BDHS obtained vaccination coverage data in 2017–2018 using two methods namely immunization cards provided to interviewers and from mothers' verbal remarks (48, 49). In this study, wealth index categorized into three variables where Richer and Richest converted to “Rich” and Poorer and Poorest to “Poor.” The interviewers transcribed the vaccination dates straight into the questionnaire if the cards were available. The respondents were asked to recollect the immunizations administered to their child if there was no vaccination card for the child or if a vaccine was not mentioned as administered in the vaccination card (8, 31, 35, 50–52). Mother's/household head's occupation is categorized as agricultural/skilled worker (farming/agricultural work and semi-skilled labor/service), household/unskilled worker (unskilled labor, home-based manufacturing, domestic service, and other), and industrial worker (professional/technical, business, factory work or blue-collar service, poultry, or cattle raising).

The weight at birth was classified as low if the weight of the child was <2.5 kg and normal if >2.5 kg. The z-scores of three anthropometric indices namely height-for-age, weight-for-age, and weight-for-height were used to assess a child's nutritional status as suggested by WHO (9). The z-score indicates how far a given result deviates from the mean, and it is commonly used to normalize data. The z-score is used in this study to compare stunting and wasting in children under-5 years by gender and age group. If a child's weight-for-height z-score is <-2, it is labeled as wasted, weight-for-age z-score is <-2, it is labeled as underweight, and the z-score of height-for-age <-2 is classified as stunted.

### Statistical analyses

To illustrate the distribution of variables, descriptive statistics were utilized whereas numbers and percentages were used for categorical variables. We used chi-square tests to identify factors associated with ARI in children, and *P* < 0.05 was considered statistically significant. We fit the design-based binary logistic regression (53) to assess the association between child ARI and types of cooking fuel in a household. For the adjusted association, the model was adjusted for the type of cooking fuel, type of roof material, child's age (months), and sex of the child. The COR and AOR were calculated and reported with the 95% CI and *p*-values (54). The specified predictor variables are used in multiple logistic regression. A survey package in R was used to conduct the statistical analyses and data management in this study. (55).

### Variable selection

The variables were selected using the Rao and Scott chi-squared test (a design-adjusted variation of the Pearson chi-squared test) (56) which was employed to account for the data's cluster-design effect. In total, 13 variables were significant with ARI at *p* < 0.05 (Table 1). Bivariate and multivariable logistic regression were carried out independently for each of the selected variables. A cutoff value of 4.00 was used as the variance inflation factor (VIF) value to analyze multicollinearity in the final model (Table 2). The area under the curve (AUC) of the receiver operating characteristic curve is used to verify the prediction accuracy of the final model (Table 3). We also utilized the Hosmer and Lemeshow goodness-of-fit test to examine the overall fit of the final model (Table 3).

**Table 1:** Frequency distribution (weighted^*^) of ARI among children younger than 5 years in Bangladesh.

**Factors**	**ARI**		
	**Yes, N (%)**	**No, N (%)**	**P-value**
**Total**	**250 (3.00)**	**8,071 (97.00)**	
**Place of residence**			
Urban	57 (2.56)	2,186 (97.44)	0.188
Rural	192 (3.17)	5,885 (96.83)	
**Region of the country**			
Barisal	19 (4.16)	443 (95.84)	<0.001
Chittagong	48 (2.76)	1,698 (97.24)	
Dhaka	42 (1.97)	2,070 (98.03)	
Khulna	13 (1.74)	754 (98.26)	
Mymensingh	17 (2.38)	688 (97.62)	
Rajshahi	38 (3.94)	933 (96.06)	
Rangpur	53 (5.98)	826 (94.02)	
Sylhet	20 (2.95)	658 (97.05)	
**Media accessibility**			
Yes	139 (3.65)	3,661 (96.35)	0.002
No	80 (2.26)	3,461 (97.74)	
**Source of drinking water**			
Piped water	5 (1.12)	481 (98.88)	0.089
Tube well	206 (3.11)	6,423 (96.89)	
Other	7 (3.13)	219 (96.87)	
**Toilet facility**			
Improved	43 (2.01)	2,121 (97.99)	0.002
Unimproved	175 (3.39)	5,001 (96.61)	
**Type of cooking fuel**			
Clean fuel	30 (1.95)	1,512 (98.05)	0.027
Solid fuel	189 (3.25)	5,603 (96.75)	
**Wealth index**			
Rich	74 (2.27)	3,201 (97.73)	0.001
Middle	40 (2.56)	1,529 (97.44)	
Poor	135 (3.89)	3,341 (96.11)	
**Electricity accessibility**			
No	56 (4.11)	1,315 (95.89)	0.023
Yes	162 (2.72)	5,808 (97.28)	
**Type of flooring material**			
Natural	155 (3.35)	4,472 (96.65)	0.091
Rudimentary	2 (2.80)	68 (97.20)	
Finished	62 (2.33)	2,582 (97.67)	
**Type of roof material**			
Natural	1 (2.69)	50 (97.31)	0.008
Rudimentary	1 (27.26)	2 (72.74)	
Finished	217 (2.97)	7,071 (97.03)	
**Type of wall material**			
Natural	24 (3.88)	605 (96.12)	0.042
Rudimentary	17 (5.48)	298 (94.52)	
Finished	177 (2.77)	6,220 (97.23)	
**Number of household member**			
Below median	80 (2.94)	2,653 (97.06)	0.855
Above median	169 (3.03)	5,418 (96.97)	
**Child's age (months)**			
24–59	112 (2.29)	4,753 (97.71)	<0.001
12–23	69 (4.09)	1,611 (95.91)	
0–11	70 (3.92)	1,706 (96.08)	
**Sex of child**			
Male	155 (3.58)	4,185 (96.42)	0.003
Female	95 (2.38)	3,886 (97.62)	
**Vaccination**			
Yes	43 (3.33)	1,252 (96.67)	0.014
No	3 (0.77)	328 (99.23)	
**Birth order**			
1–3	210 (2.88)	7,074 (97.12)	0.074
4–6	39 (4.04)	935 (95.96)	
6+	1 (0.98)	62 (99.02)	
**Place of delivery**			
Home	93 (3.62)	2,468 (96.38)	0.337
Hospital	79 (3.09)	2,472 (96.51)	
**Weight at birth**			
Low	21 (3.03)	677 (96.97)	0.762
Normal	53 (3.29)	1,572 (96.71)	
**Delivery by C-section**			
Yes	49 (2.86)	1,656 (97.14)	0.208
No	123 (3.61)	3,278 (96.39)	
**Season of birth**			
Summer	73 (3.40)	2,077 (96.60)	0.051
Autumn	46 (2.46)	1,829 (97.54)	
Winter	75 (3.70)	1,942 (96.30)	
Spring	56 (2.45)	2,224 (97.55)	
**Medication for intestinal parasites**			
No	168 (3.41)	4,759 (96.59)	0.145
Yes	82 (2.41)	3,301 (97.59)	
**Vitamin A supplementation**			
No	66 (2.96)	2,170 (97.04)	0.883
Yes	184 (3.03)	5,879 (96.97)	
**Stunting**			
No	159 (2.94)	5,258 (97.06)	0.217
Yes	85 (3.54)	2,316 (96.46)	
**Wasting**			
No	221 (3.09)	6,924 (96.91)	0.496
Yes	24 (3.60)	635 (96.40)	
**Underweight**			
No	223 (3.04)	7,099 (96.96)	0.203
Yes	23 (4.07)	536 (95.93)	
**Mother's age group (in years)**			
15-24	126 (3.19)	3,824 (96.81)	0.722
25-34	105 (2.81)	3,613 (97.19)	
45+	19 (2.97)	634 (97.03)	
**Mother's education level**			
Secondary or higher	29 (2.18)	1,287 (97.82)	0.221
Primary	127 (3.14)	3,920 (96.86)	
No Education	94 (3.18)	2,864 (96.82)	
**Mother's BMI**			
Obese	10 (2.07)	477 (97.93)	0.719
Overweight	54 (3.20)	1,624 (96.80)	
Normal Weight	150 (3.07)	4,734 (96.93)	
Underweight	34 (3.04)	1,093 (96.96)	
**Number of living children**			
≤2	178 (3.00)	5,751 (97.00)	0.990
3–4	62 (3.03)	1,994 (96.97)	
5+	10 (2.88)	326 (97.12)	
**Mother's occupation**			
Agriculture	87 (3.64)	2,314 (96.36)	0.026
Don't work	121 (2.52)	4,670 (97.42)	
Industires	42 (3.72)	1,085 (96.28)	
**Mother's work for**			
Family	88 (4.05)	2,077 (94.95)	0.111
Else	15 (2.23)	641 (97.77)	
Self	27 (3.83)	677 (96.17)	
**Household head's occupation**			
Agriculture	62 (3.73)	1,590 (96.27)	0.185
Don't work	4 (1.95)	181 (98.05)	
Industries	185 (2.86)	6,283 (97.14)	
**Household head's education**			
Secondary or higher	25 (1.70)	1,436 (98.30)	0.017
Primary	85 (3.15)	2,600 (96.85)	
No education	137 (3.40)	3,892 (96.60)	
**Type of household head's education**			
School	216 (3.10)	6,766 (96.90)	0.969
Madrasha	23 (3.13)	721 (96.87)	

* Frequencies are weighted using sample weight.

**Table 2:** Generalized variance inflation (GVIF) value of the final model of ARI among under-5 years children in Bangladesh.

**Variables**	**Degrees of freedom (Df)**	**GVIF**
Region of the country	7	1.31
Media accessibility	1	1.04
Toilet facility	1	1.01
Type of cooking fuel	1	1.01
Wealth index	2	1.03
Electricity accessibility	1	1.03
Type of roof material	2	1.02
Type of wall material	2	1.01
child's age (months)	2	1.02
Sex of Child	1	1.02
Vaccination	1	1.01
Mother's occupation	2	1.02
Household head's occupation	2	1.03

**Table 3:** Test for the goodness of fit and predictive accuracy of the final model.

**Hosmer and Lemeshow goodness of fit test**		
**Value**	**Df**	**P-value**
8.24	24	0.76
Area under the curve (AUC) of the receiver operating characteristic curve (ROC)		
Value	0.61

## Results

### Study sample characteristics

A total number of 20,160 households were eligible for the interview although 20,250 houses were approached. Floodwater undermined three clusters, resulting in the loss of 90 households. As such, 20,160 households with 8,347 children were enrolled in the study. Of those enrolled, 26 children were eliminated due to visitors and non-surviving children. Finally, 8,321 observations were obtained for conducting this study (Figure 1).

### Socio-demographic characteristics

In the public health sector, despite many challenges in socio-demographic conditions, Bangladesh coined huge success from the liberation of 1971, decreasing pregnancy and maternal deaths (39). Table 1 presents the results of the chi-square analysis for identifying household factors associated with ARI. The results of the chi-square analysis indicate the regional distribution, media accessibility, toilet facility, types of cooking fuel, wealth index, electricity accessibility, types of roof material, and types of wall material are statistically significant (*p* < 0.05) factors. Among 8,321 children, 73.04% were from rural areas and 26.96% from urban areas. By region of the country, 25.38% were from Dhaka, 20.99% from Chittagong, 11.67% from Rajshahi, 10.56% from Rangpur, 9.23% from Khulna, 8.48% from Mymensingh, 8.15% from Sylhet, and 5.55% from Barisal. We also found that 51.76% of the household has media accessibility and 48.24% does not have any type of media accessibility in their household. Furthermore, 90.30% used tube well sources of water, 6.63% of respondents used piped sources of water, and 3.07% used other sources of water. From our results, we observed that 70.52% has unimproved toilet facilities, and 29.48% had improved toilet facilities. According to wealth status, 41.77% are from the poorest households, 44.68% were from the 25–34 years age group and 7.86% were from the 45 years or above age group. By wealth status, 41.77% are from the lower wealth category families, 39.36% are children from higher wealth category families, and 18.86% are from middle-class families. About 78.97% used solid cooking fuel, and 82.32% has electric accessibility.

Most mothers (79.68%) of the children were from the 15–24 years age group, 44.68% were from the 25–34 years age group, and 7.86% were from the ≥45 years age group. A large group of mothers (79.68%) was vaccinated, whereas a few (20.32%) were not. As for parents' education characteristics, 48.64% of respondents had primary education, 15.81% had secondary or higher education, and 35.55% of respondents were uneducated. According to mothers' education status, 77.89% of household heads were industrial workers, 19.89% were agricultural workers, and only 2.22% were unemployed. Among all household heads, 49.29% of household heads had no education (Table 1). In total, 58.47, 20.19, and 21.34% of children were included from the 24–59 months, 12–23 months, and 0–11 months age groups, respectively. There were 52.16% of male children, and 30.72% were stunted. The birth order distribution of children was 87.5% in the 1–3 group, 59.3% were born at a health facility, 69.95% were delivered at a normal weight, and 66.61% and 47.84% were female children delivered by normal delivery (Table 1).

### Relationship between the prevalence of ARI and other confounding factors

Table 1 shows the relationship between the incident of ARI with different household, maternal, and child characteristics. From our chi-square test, region of the country, media accessibility, toilet facility, type of cooking fuel, wealth index, electricity availability, roof materials type, vaccination, mothers' occupation, education of household head, child age, and sex of the child showed a statistically significant effect on ARI (*P* < 0.05). This study found that the highest prevalence (5.98%) of ARI was observed in children living in Rangpur–those who were infected by ARI in the last 2 weeks of the survey, and was the lowest (1.74%) in Khulna. ARI depends on the Child's household with media accessibility. Children who live with media access had ARI at a rate of 3.65% but those children who live without media accessibility (2.26%) had lower ARI. Toilet facility and wealth index significantly influence ARI diseases. The proportion of getting ARI among children and households with poor and unhygienic toilets was 3.39% whereas with hygienic toilets was 2.01%. The proportion of exposure to ARI was relatively lower (2.27%) in children with a high family wealth index compared to the middle or poor. This infers wealthy families spend more money to improve the health and nutritional standards of their children and acquire clean energy sources for cooking and heating purposes, thus reducing exposure to ARI.

### Biomass fuel consumption at the household level

**Figure 2:**
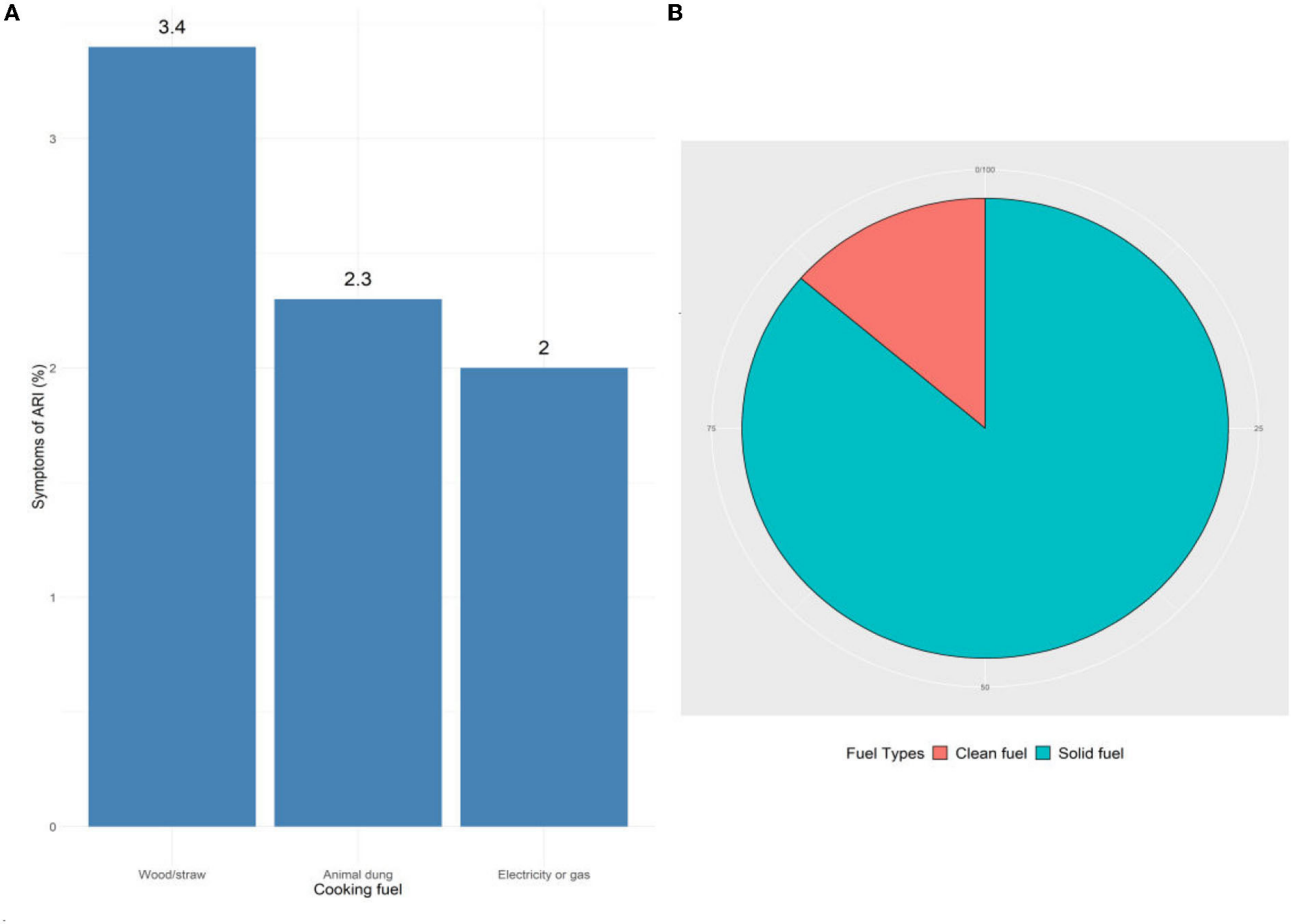
Fuel types used in ARI-infected children's household in the study area. **(A)** All fuel types, **(B)** clean and solid fuel.

According to Aziz et al. (57), about 19% of households used clean cooking while WHO guidelines described proper fuel usage without much air pollution (58). The wider access to clean cooking systems for all people by 2030 is one of the important targets in the Sustainable Development Goal (SDG) 7. In total, 86.3% of households consume solid fuels whose children suffer from ARI–among them, 3.4% use wood/straw, and 2.3% use animal dung (Figure 2). Currently, 2.7 billion households lack access to use clean cooking around the world, while the maximum number of people uses solid fuels, including charcoal, peat, wood, coal, corn, rye, wheat, and other grains.

**Figure 3:**
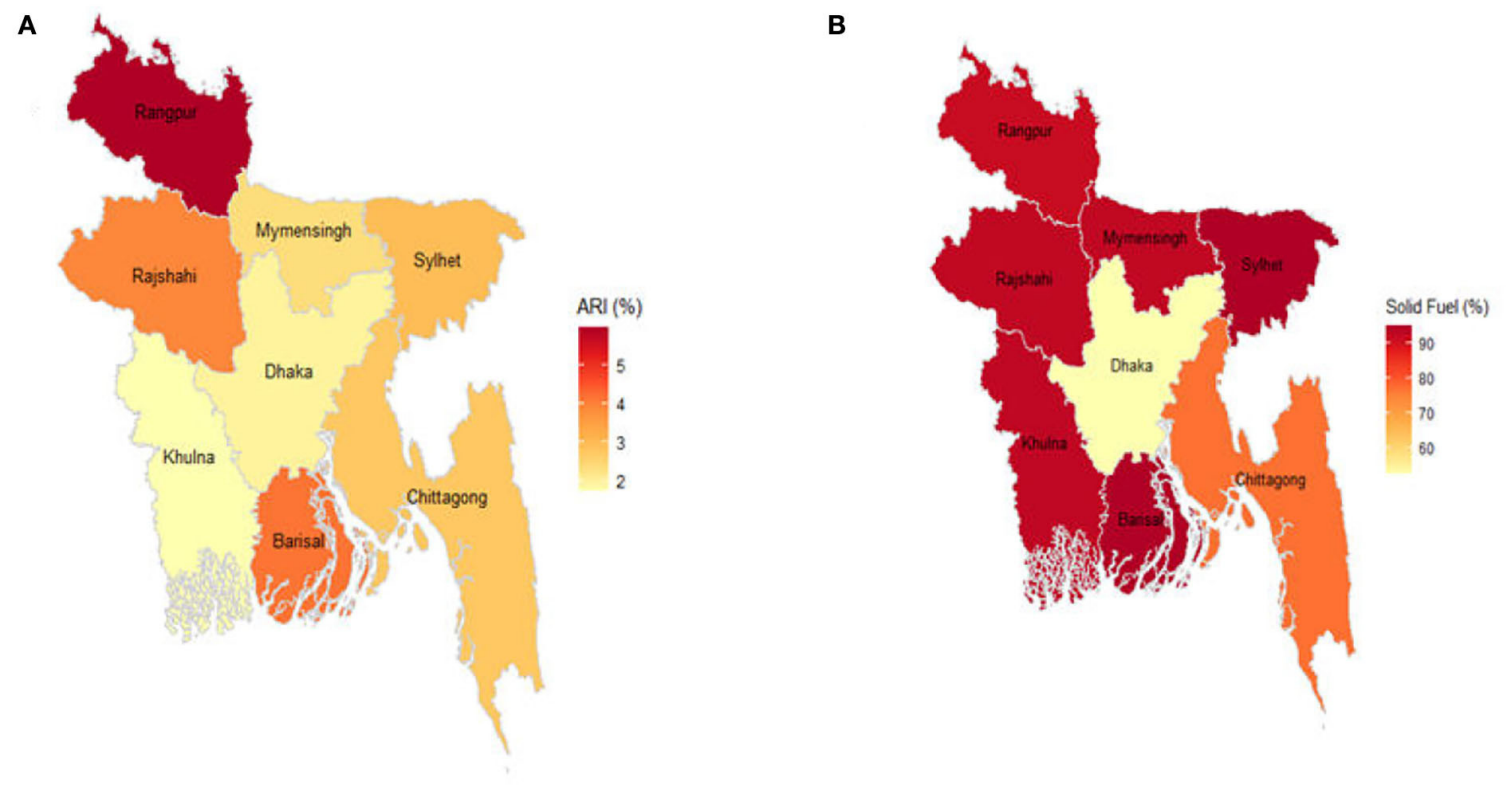
Prevalence of ARI and solid fuel (%) in different regions of Bangladesh. **(A)** ARI. **(B)** Solid fuel.

Solid fuel is found in a variety of forms and is comparatively easier to use and cheaper but heavier to transport than liquid fuel. The Rangpur and Barisal regions of Bangladesh have the highest prevalence of solid fuel consumption, whereas the Dhaka region has the lowest prevalence (Figure 3). The northern division of Bangladesh Rangpur shows the maximum ARI infection rate, while the capital of Bangladesh Dhaka and the southern part of Khulna reveals the lowest infection with ARI. The results show the ARI infection rate is moderate in Rajshahi and Barisal.

According to the results of the Rao-Scott chi-squared independence test, the prevalence of ARI is significantly associated (*P* < 0.05) with the type of fuel used in the home (Table 1).

### Model evaluation

**Figure 4:**
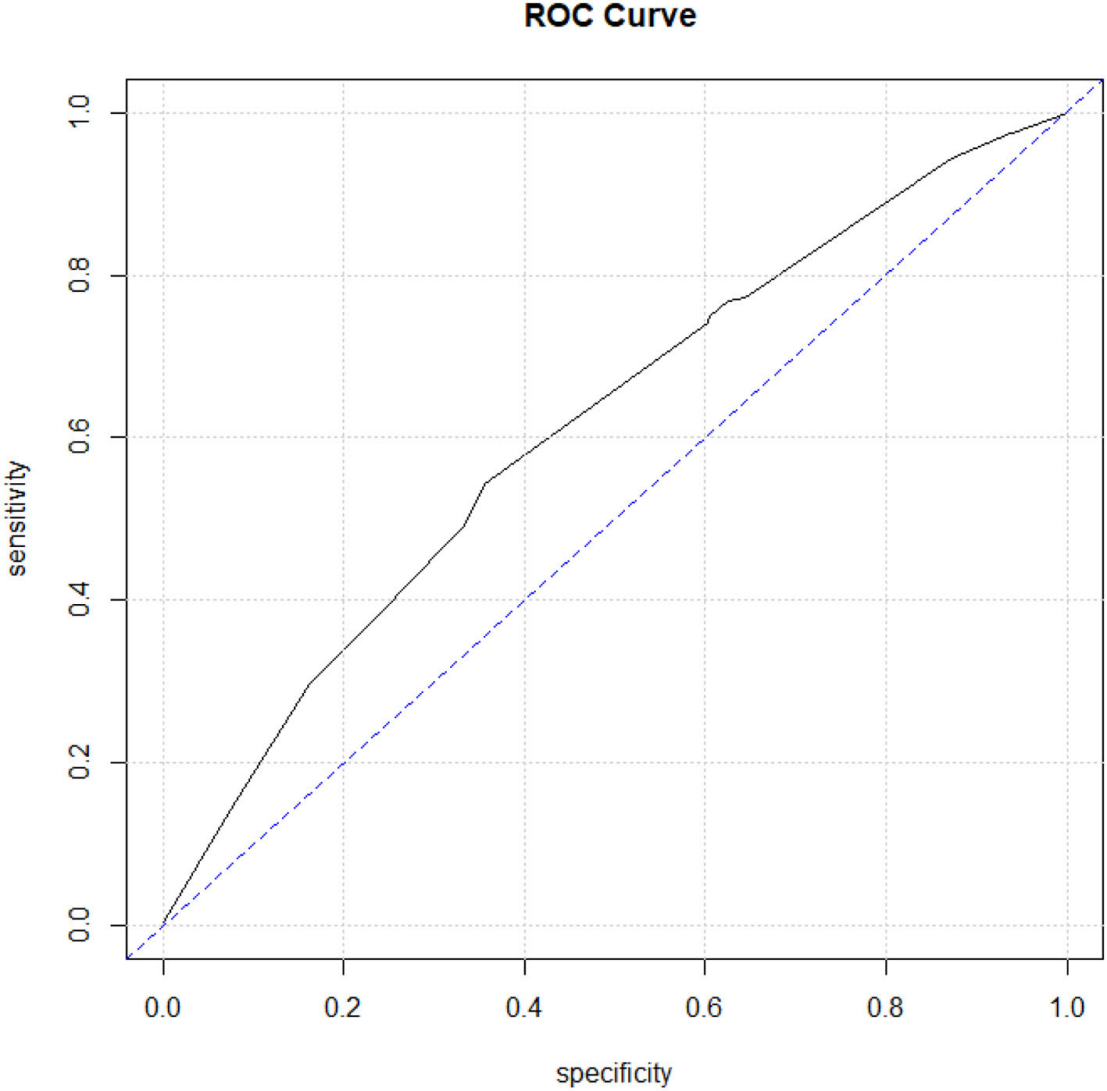
Model evaluations using the receiver operating characteristic curve (ROC curve). X-axis indicates specificity, whereas Y-axis indicates sensitivity.

The VIF results demonstrated no multicollinearity in the final multivariable logistic model (Table 2). The classification accuracy is acceptable with an AUC value of 0.61. The model also passed the Hosmer and Lemeshow goodness-of-fit test (value = 8.2419, degrees of freedom = 8, *P* = 0.760), indicating no lack of fit (Tables 2, 3; Figure 4).

### Association between the prevalence of ARI and solid fuel

According to (18), about 50,320 (4.9%) child deaths are attributed annually to fuel in Ethiopia (28). This cross-section-based research study also proved that biomass fuel and ARI were correlated in 422 households in the slum of Addis Ababa from January to February 2012. Table 4 demonstrates the crude and adjusted association between household fuel use and ARI among children under-5 years in Bangladesh. In crude analysis, the solid fuel risk group in household fuel type is associated with 1.69 times higher odds of ARI than the clean fuel risk group (COR: 1.69; 95% CI: 1.06–2.71). After adjusting the model for potential confounders and risk factors, we observe 1.69 times the odds of ARI among those children from the solid fuel risk group in households than those from clean fuel (AOR: 1.69; 95% CI: 1.05–2.72) (Table 4).

**Table 4:** Association between the household type of fuel use and ARI among under-5 years children in Bangladesh.

		**Unadjusted model**			**Adjusted model^a^**		
**Variables**		**COR**	**95% CI**	**P-value**	**AOR**	**95% CI**	**P-value**
**Type of cooking fuel**	Solid fuel	1.69	[1.06, 2.71]	0.028	1.69	[1.05, 2.72]	0.030
	Clean fuel	1			Ref.		

COR, crude odds ratio; AOR, adjusted odds ratio; CI, confidence interval; Ref, reference.

a The familiar analysis was performed using the design-based binary logistic regression, adjusted for regions of the country, media accessibility, toilet facility, type of cooking fuel, wealth index, electricity accessibility, type of roof material, type of wall material, child's age (months), sex of child, vaccination, mother's occupation, and household head's occupation.

## Discussion

Human respiratory-related infections (RTIs) are known as the most familiar human infections, while ARIs are found in the upper and lower side of respiratory tract. The population compactness of Bangladesh is 1,265 sq/km with a total of 165 million as of 04 July 2022 (56, 59). Of the study households, solid fuel (i.e., coal/lignite, charcoal, wood, straw/shrubs/grass, agricultural crops, and animal dung) is the most widely used fuel for cooking in Bangladesh. Biomass burning inside homes generates high-magnitude concentrations of harmful substances, such as CO (an 8-h average: about 40.7 ppm SD: 40.0 ppm), PM 2.5, and PM 10, exceeding WHO Indoor Air Quality Standards (57). This stimulates an inflammatory response in the airways (60) and greater tissue damage favoring the development of respiratory diseases (61).

These findings show an association between solid fuel consumption in households and ARI episodes in children under-5 years old, which is consistent with previous studies (62). A few studies, however, have failed to establish a relationship between smoke and ARI (63). A follow-up study in Kenya developed an exposure-response function between exposure to particles from biomass fuel combustion and ARI (21). The prevalence of ARI is greater in children who live in households that use solid fuel. A national representative sample of a Bangladesh Urban Health Survey conducted in 2013 reported 39.5% of solid fuel users in urban areas while 60.5% as clean fuel users (64). More than half of households in India and Nepal use 54% (2015–2016) and 66% (2016) solid fuel for cooking.

In our study, we observe that ARI is more frequent in the case of children of uneducated mothers. According to a study conducted by Johns Hopkins University's Department of International Health, most mothers believe that a “wind-carrying sickness” may kill their children, but ARI is deemed to be more controllable, and this is due to illiteracy (65). The government's effort to educate girls beyond the secondary level is a requirement to improve future health literacy and childcare management. In this instance, the government's free primary and secondary education program is extremely important, and it should be strengthened and expanded (66). This is likely because mothers who use vaccination programs are more aware of healthcare facilities and are more likely to seek out early consultation for children's illnesses, which may perhaps curb morbidity and mortality (67).

Our findings show that women in older age cohorts and children with higher birth order have a lower prevalence of ARI. This can be due to childcare expertise and experience acquired by older women, which is unmistakably advantageous over younger women (66). Household wealth is defined according to the respondent's reported household assets, which were assigned a standardized score and categorized into three categories, namely lower, middle, and higher. Being overweight or obese (BMI ≥ 25 kg/m^2^) is a rising issue connected with the risk of ARIs (68).

In our study, the incidence of ARI was found superior in stunted infants, wasted infants, and infants with low birth weight. Stunting is linked to a long period spent in poor environmental circumstances alongside a low socioeconomic level as a child (69). ARI bouts that are more frequent and long-lasting may cause growth retardation. Moreover, other nutritional disorders were also found to be associated with ARI. Malnutrition was found to be strongly related to ARI in this investigation, as it has been in prior studies (19). A study in the Philippines included age-stratified risks in children ≤ 23 months of age and reported the highest risk of deaths from ARI due to malnutrition among those aged 12–22 months (18). A study conducted in New Delhi, India, exposed severe malnutrition as the forecaster of mortality in ARI in 2-week to 5-year-old children. Overall, malnutrition is associated with a 2- to 3-fold increase in ARI mortality (70).

The studies differ in terms of the plan, exposure dimension, and result evaluation, and the recent findings concerning the relationship between solid fuel consumption and the occurrence of acute respiratory diseases are parallel to most research findings in India (OR 4.0, 95% CI 2.0–7.9), Zimbabwe (OR 2.1, 95% CI 1.5–3.1), Nepal (OR 2.3, 95% CI 1.8–2.9), Gambia (OR 5.2, 95% CI 1.7–15.9), and a meta-analysis-based research studies (OR 2.3, 95% CI 1.9–2.7) (17–23). The safe fuel and adapting to it necessitating significant behavioral changes and other factors must be considered as part of broader initiatives, such as reforming kitchen structure, upgrading knowledge, as well as boosting the adequate and satisfactory level of awareness among household family members and broader communities, which can facilitate lowering the risk of air pollution and concomitant childhood ARI prevalence.

## Strengths and limitations

This is the first research that assesses the association between exposure to solid fuel and ARI episodes in children aged under-5 years in Bangladesh. We used a sufficiently large nationally representative dataset that reflects Bangladesh's whole population. We also considered a wide range of factors that influence the public's knowledge of the issue. We also looked at model-fitting criteria, which were mostly absent in the literature. Despite this, there were certain limitations to our research. Because we used secondary data, we had no control over the variable selection, data quality, or measurement indication. Besides, environmental and behavioral factors were missing, which are important in exposure assessment. Furthermore, the fluctuating economic performance in Bangladesh could have shifted the level of fuel used among households.

## Conclusion

This study shows that solid fuel is significantly associated with an increased risk of ARI in children under the age of five in Bangladesh, which underscores the demand for clean and alternative cooking fuels to reduce the occurrences of ARI diseases, especially in rural and poor households. Even though such association requires further investigation using more specific measures of exposure to smoke and clinical measures of ARI, this study has crucial implications for the preventative measures of ARI among children in Bangladesh as a significant portion of households use solid fuel for cooking in the country's rural areas. Short-term measures such as using efficient burners and instructing mothers about keeping children away while cooking might help lower the prevalence of ARI and accompanying morbidity and death. Long-term interventions for shifting to cleaner and safer fuels could be established, which may include infrastructural and economic progress. Our study also shows that ARI, which is currently prevalent on a wide scale, is on the rise. Hence, the government could devote high attention to ARI control and prevention as a top priority.

## Data availability statement

The original contributions presented in the study are included in the article/Supplementary material, further inquiries can be directed to the corresponding authors.

## Author contributions

AA: formal analysis and writing—original draft. AM and SM: data curation. MS and SM: writing and reviewing. SM: methodology. KS, AM, AA, and KD: reviewing and editing. All authors contributed to the article and approved the submitted version.

## Funding

This study was funded by KTH Royal Institute and supported by President Abdul Hamid Medical College Hospital, Kishoreganj, Bangladesh.

## Conflict of interest

The authors declare that the research was conducted in the absence of any commercial or financial relationships that could be construed as a potential conflict of interest.

## Publisher's note

All claims expressed in this article are solely those of the authors and do not necessarily represent those of their affiliated organizations, or those of the publisher, the editors and the reviewers. Any product that may be evaluated in this article, or claim that may be made by its manufacturer, is not guaranteed or endorsed by the publisher.
