# Corrigendum: Association of household fuel with acute respiratory infection (ARI) under-five years children in Bangladesh

**DOI:** 10.3389/fpubh.2023.1299521

**Published:** 2023-10-10

**Authors:** Md. Aminul Islam, Mohammad Nayeem Hasan, Tanvir Ahammed, Aniqua Anjum, Ananya Majumder, M. Noor-E-Alam Siddiqui, Sanjoy Kumar Mukharjee, Khandokar Fahmida Sultana, Sabrin Sultana, Md. Jakariya, Prosun Bhattacharya, Samuel Asumadu Sarkodie, Kuldeep Dhama, Jubayer Mumin, Firoz Ahmed

**Affiliations:** ^1^COVID-19 Diagnostic Lab, Department of Microbiology, Noakhali Science and Technology University, Noakhali, Bangladesh; ^2^Advanced Molecular Lab, Department of Microbiology, President Abdul Hamid Medical College, Karimganj, Bangladesh; ^3^Department of Statistics, Shahjalal University of Science and Technology, Sylhet, Bangladesh; ^4^Joint Rohingya Response Program, Food for the Hungry, Cox's Bazar, Bangladesh; ^5^Department of Applied Chemistry and Chemical Engineering, Noakhali Science and Technology University, Noakhali, Bangladesh; ^6^Department of Banking and Insurance, University of Chittagong, Chittagong, Bangladesh; ^7^Department of Environmental Science and Management, North South University, Bashundhara, Dhaka, Bangladesh; ^8^COVID-19 Research, Department of Sustainable Development, Environmental Science and Engineering, KTH Royal Institute of Technology, Stockholm, Sweden; ^9^Nord University Business School (HHN), Bodø, Norway; ^10^Division of Pathology, ICAR-Indian Veterinary Research Institute, Bareilly, Uttar Pradesh, India; ^11^Platform of Medical and Dental Society, Dhaka, Bangladesh

**Keywords:** developing countries, solid fuels, clean fuels, under-five children, acute respiratory infection (ARI)

In the published article, there was an error in affiliation 6. Instead of “School of Accounting and Finance, University of Greenwich, London, United Kingdom”, it should be “Department of Banking and Insurance, University of Chittagong, Chittagong, Bangladesh”.

The authors apologize for this error and state that this does not change the scientific conclusions of the article in any way. The original article has been updated.

