# Supplementary material for: Association of household fuel with acute respiratory infection (ARI) under-five years children in Bangladesh
Source: Front Public Health. 2022 Dec 1;10:985445. doi: 10.3389/fpubh.2022.985445 (PMC9752885; doi:10.3389/fpubh.2022.985445)
Supplement: Supplementary file 1 [file Table_1.docx]

**Supplementary files**

**Table 1:** Factors associated with ARI among children younger than 5 years in Bangladesh

| Factors | Unadjusted model | | Adjusted model | |
| --- | --- | --- | --- | --- |
|  | COR (95% CI) | P-value | COR (95% CI) | P-value |
| **Region of the country** | | | | |
| Barisal |  |  |  |  |
| Chittagong | 0.65 [0.37, 1.14] | 0.134 | 0.58 [0.12, 2.85] | 0.499 |
| Dhaka | 0.46 [0.27, 0.81] | 0.007 | 0.32 [0.06, 1.60] | 0.165 |
| Khulna | 0.41[0.20, 0.83] | 0.015 | 0.13[0.01, 1.44] | 0.096 |
| Mymensingh | 0.56 [0.31, 1.02] | 0.057 | 1.17 [0.24, 5.65] | 0.848 |
| Rajshahi | 0.95 [0.56, 1.60] | 0.837 | 0.72[0.12, 4.25] | 0.712 |
| Rangpur | 1.47 [0.89, 2.41] | 0.129 | 1.48 [0.23, 9.62] | 0.679 |
| Sylhet | 0.70[0.41,1.21] | 0.203 | 0.77 [0.17, 3.53] | 0.732 |
| **Media accessibility** | | | | |
| Yes | 0.61[0.44, 0.84] | 0.002 | 0.65 [0.19, 2.21] | 0.491 |
| No |  |  |  |  |
| **Toilet facility** | | | | |
| Improved |  |  |  |  |
| Unimproved | 1.71[1.21, 2.43] | 0.003 | 1.15 [0.30, 4.35] | 0.841 |
| **Type of cooking fuel** | | | | |
| Clean fuel |  |  |  |  |
| Solid fuel | 1.69 [1.06, 2.71] | 0.028 | 1.69 [1.05, 2.72] | 0.030 |
| **Wealth index** | | | |  |
| Rich |  |  |  |  |
| Middle | 1.13[0.73, 1.74] | 0.578 | 1.39[0.19, 9.86] | 0.586 |
| Poor | 1.74 [1.26, 2.40] | 0.001 | 1.55 [0.32, 7.54] | 0.534 |
| **Electricity accessibility** | | | | |
| No |  |  |  |  |
| Yes | 0.65 [0.45, 0.95] | 0.024 | 0.63[0.34, 0.98] | 0.137 |
| **Type of roof material** | | | | |
| Natural |  |  |  |  |
| Rudimentary | 13.55[1.63, 22.44] | 0.016 | 15.11[5.21, 17.99] | 0.022 |
| Finished | 1.11 [0.25, 4.97] | 0.894 | 1.97 [0.80, 4.81] | 0.211 |
| **Type of wall material** | | | | |
| Natural |  |  |  |  |
| Rudimentary | 1.44 [0.63, 3.25] | 0.382 | 1.23[0.25, 5.96] | 0.795 |
| Finished | 0.71 [0.43, 1.17] | 0.174 | 0.34 [0.12, 0.77] | <0.001 |
| **Child's age (months)** | | | | |
| 24-59 |  |  |  |  |
| 12-23 | 1.82 [1.32, 2.49] | <0.001 | 1.29 [0.43, 3.90] | 0.653 |
| 0-11 | 1.74[1.24, 2.43] | <0.001 | 1.62 [0.56, 4.65] | 0.372 |
| **Sex of Child** | | | | |
| Male |  |  |  |  |
| Female | 0.65 [0.50, 0.87] | 0.003 | 0.54 [0.25, 1.16] | 0.113 |
| **Vaccination** | | | | |
| Yes | 0.22 [0.06, 0.82] | 0.024 | 0.21 [0.05, 0.91] | 0.037 |
| No |  |  |  |  |
| **Mother’s occupation** | | | | |
| Agriculture |  |  |  |  |
| Don’t work | 0.68 [0.49, 0.94] | 0.022 | 0.63 [0.11, 3.45] | 0.591 |
| Industires | 1.02 [0.67, 1.57] | 0.915 | 1.13 [0.48, 2.68] | 0.772 |
| **Household head’s occupation** | | | | |
| Agriculture |  |  |  |  |
| Don’t work | 0.51 [0.15, 1.70] | 0.275 | 0.84 [0.11, 6.34] | 0.864 |
| Industries | 0.76 [0.55, 1.05] | 0.099 | 0.89 [0.37, 2.10] | 0.788 |
